# The Pleiotropic Function of Human Sirtuins as Modulators of Metabolic Pathways and Viral Infections

**DOI:** 10.3390/cells10020460

**Published:** 2021-02-21

**Authors:** Mohammed Hamed Alqarni, Ahmed Ibrahim Foudah, Magdy Mohamed Muharram, Nikolaos E. Labrou

**Affiliations:** 1 Department of Pharmacognosy, College of Pharmacy, Prince Sattam Bin Abdulaziz University, Alkharj 11942, Saudi Arabia; a.foudah@psau.edu.sa; 2 Department of Pharmaceutics, College of Pharmacy, Prince Sattam Bin Abdulaziz University, Alkharj 11942, Saudi Arabia; m.moharm@psau.edu.sa; 3 Department of Microbiology, College of Science, Al-Azhar University, Nasr City, Cairo 11884, Egypt; 4 Laboratory of Enzyme Technology, Department of Biotechnology, School of Applied Biology and Biotechnology, Agricultural University of Athens, 75 Iera Odos Street, GR-11855 Athens, Greece

**Keywords:** Acetylation, antiviral, COVID-19, infection, metabolism, NAD^+^, sirtuins, SIRT1, virus

## Abstract

Sirtuins (SIRTs) are nicotinamide adenine dinucleotide-dependent histone deacetylases that incorporate complex functions in the mechanisms of cell physiology. Mammals have seven distinct members of the SIRT family (SIRT1-7), which play an important role in a well-maintained network of metabolic pathways that control and adapt the cell to the environment, energy availability and cellular stress. Until recently, very few studies investigated the role of SIRTs in modulating viral infection and progeny. Recent studies have demonstrated that SIRT1 and SIRT2 are promising antiviral targets because of their specific connection to numerous metabolic and regulatory processes affected during infection. In the present review, we summarize some of the recent progress in SIRTs biochemistry and their emerging function as antiviral targets. We also discuss the potential of natural polyphenol-based SIRT modulators to control their functional roles in several diseases including viral infections.

## 1. Introduction

Human SIRTs form an evolutionarily conserved family of proteins that are ubiquitously expressed in all taxa. SIRTs are classified as NAD^+^ dependent deacylases/mono-ADP ribosyltransferases that regulate numerous cellular functions, including metabolism, cell cycle, stress and longevity [1,2,3,4]. Their name comes from the first member of the family that was studied in Saccharomyces cerevisiae, which was characterized as “silent information regulator 2” (Sir2), because it appeared to be involved in gene transcription silencing [5]. Subsequent studies have confirmed that the enzymatic activity of SIRTs affects the expression of various genes through the deacetylation of the ε-terminus of the amino acid Lys residues of histones. The discovery of SIRT isoforms in yeast and later in bacteria, plants and mammals suggests that Sir2 belongs to a very large and ancient family of genes [6,7]. 

Early studies have established that SIRTs affect the lifespan of organisms. Indeed, not only in yeast but also in other organisms, such as *Drosophila melanogaster* and *Caenorhabditis elegans*, expression of SIRTs leads to a longer shelf life, following a mechanism similar to that of calorie restriction [8,9,10]. Under calorie restriction conditions, SIRTs are able to increase mitochondrial biogenesis, enhance metabolism [11] and reduce the levels of reactive oxygen species (ROS), thus alleviating the progression of inflammation [12,13,14,15,16]. These observations opened new avenues in understanding the mechanisms that are affected by the action of SIRTs. 

Mammals have seven distinct members of the SIRT family, SIRT1-7, which play an important role in the regulation of gene expression, mainly by controlling the posttranslational modification status of histones and transcription factors [7,17,18]. Another role that has been assigned to SIRTs is the regulation of a well-maintained network of metabolic pathways [15,17,18,19]. SIRTs were originally classified as NAD^+^-dependent histone deacetylases, indicating that these enzymes are involved in different metabolic pathways as part of the transcriptional adaptation mechanisms, acting as metabolic sensors. For example, the three mitochondrial SIRTs (SIRT3, SIRT4 and SIRT5) can control essential metabolic pathways. SIRT3 affects a range of key metabolic processes, such as fatty acid oxidation and the tricarboxylic acid (TCA) cycle [13]. SIRT4 inhibits the pyruvate dehydrogenase complex [20]. Furthermore, SIRT5 with its desuccinylase and demalonylase activity controls several metabolic pathways, including the urea cycle [21,22,23]. Considering their role in the regulation of metabolism, SIRTs were studied in the context of metabolic diseases [24,25,26,27,28]. Therefore, SIRTs regulate a large number of pathways, such as glucose and fatty acid metabolism, apoptosis, DNA repair, neuronal generation, inflammatory response and even the regulation of the circadian clock of organisms [1,2,4].

## 2. Methods

### 2.1. Data Sources

The following bibliographic databases were used:

Medline/PubMed: http://www.ncbi.nlm.nih.gov/pubmed;
Web of Science: http://www.webofknowledge.com;
SCOPUS: http://www.scopus.com; Google Scholar: https://scholar.google.com; Protein data bank: https://www.rcsb.org/.

### 2.2. Search Terms and Search Strategies

The authors searched databases until 31st January 2021. The bibliographic databases were searched using combinations of the following keywords: activator, ageing, antiviral compounds, calorie restriction, coronavirus infections, Covid-19, energy metabolism, inflammation, inhibitors, lysine acylation, metabolism, mitochondria, modulators, NAD^+^, natural products; SARS-CoV-2, Sirtuins, virus.

### 2.3. Type of Investigations

The authors searched all relevant studies including human and animal models and cell lines.

## 3. Acetylation and Deacetylation of Proteins

The conjugation of acetyl groups to proteins is a posttranslational modification mechanism that is widely used by eukaryotic cells to regulate various functions [29]. It is estimated that more than 6800 known acetylation sites exist in mammalian proteins [30]. A common and possibly more important form of acetylation is carried out post-translationally, at the ε-terminus amino group of Lys residues where an acetyl group is transferred from acetyl-CoA [31].

Lysine is a positively charged amino acid; therefore, its acetylation neutralize its positive charge and as a consequence alters the electrostatic properties of the protein [32,33]. This modification is a reversible process and takes place largely in histones, which are proteins that bind to DNA and form the basic structure of chromatin, the nucleosome. The group of enzymes that are responsible for the acetylation of histones are the histone acetyltransferase (HATs) [34]. The DNA has a negative charge while histones are rich in positively charged amino acid residues (lysine, arginine and histidine). The replication and transcription processes of DNA require the loss of interaction between DNA and histones, which is achieved by reducing the positive charge of histones. This is a reversible process, and therefore the degree of histone acetylation determines their access to DNA [32,33]. Conversely, histone deacetylation leads to transcriptional silencing of genes, as a heterochromatin environment is created around the gene, making it inaccessible to transcriptional mechanisms [35,36].

## 4. The Function, Structure and Regulation of SIRTs

### 4.1. The Human SIRT Family and Their Subcellular Status

SIRT genes are found in virtually all organisms, both prokaryotic (mycobacteria, eubacteria and archaea) and eukaryotic (yeasts and protozoa). In prokaryotes, SIRTs are encoded primarily by a single gene, while in eukaryotes by multiple genes. The variety of genes encoding these enzymes leads to the formation of isoforms, which have distinct catalytic functions and are localized in different subcellular compartments [2,18,37,38,39,40]. In mammals, the SIRT family consists of seven isoenzymes, SIRT1-7 (Table 1). Phylogenetic analysis of different SIRT genes (eukaryotic and prokaryotic) indicated that the seven mammalian SIRTs can be grouped into four different classes (I—IV) [2,6,18,38,39] (Table 1). In Class I belongs the SIRT1, SIRT2 and SIRT3 and is divided in three sub-classes: a, b and c. Class II includes SIRT4, which also includes SIRTs from bacteria, insects, nematodes, fungus and protozoans. SIRT5 is part of the Class III SIRTs, whereas Class IV includes SIRT6 and SIRT7, divided in two sub-classes IVa and IVb.

**Figure 1:**
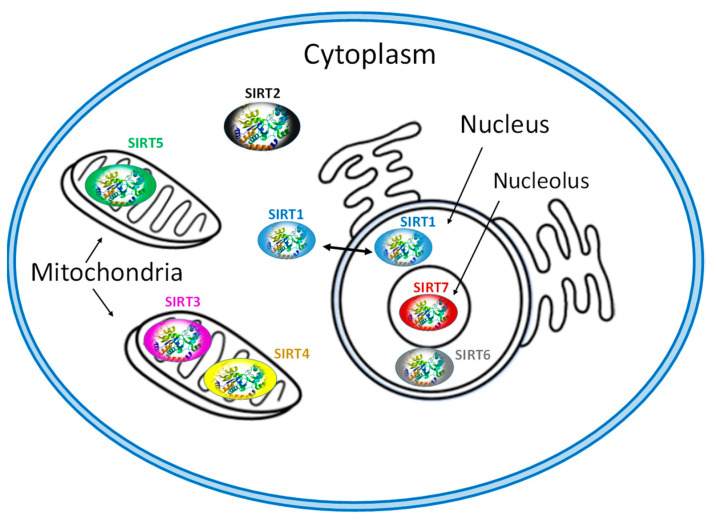
Subcellular location of seven mammalian SIRTS (SIRT1-7).

Each of SIRT isoenzymes is located in different subcellular compartments, depending on the function it performs, as shown in Figure 1. Some SIRTs can delocalize depending on the cell or tissue type, the developmental stage, metabolic status, and certain stress conditions [1,3,7,41]. The distribution of SIRTs in different subcellular compartments allows them to interact with a wide variety of transcription factors and to participate in the regulation of different metabolic processes, such as apoptosis, glucose homeostasis, insulin resistance, stress resistance, circadian rhythm, mitochondrial biogenesis, and DNA repair [1,2,3,4,43]. In addition, they play a key role in the development of inflammation and autophagy [56]. 

The isoenzymes SIRT1, SIRT6 and SIRT7 are found in the nucleus of the cell and epigenetically affect gene regulation, exerting their enzymatic activity as histone deacetylases [7,41]. SIRT1 contains the nuclear localization signal peptide sequence (KRKKRK) in 41–46 residues; however, under certain conditions, it can also be transported from the nucleus to the cytoplasm where it is involved in the regulation of insulin metabolism [41,42]. SIRT6 is found in heterochromatin and is involved in DNA repair mechanisms and also occurs in nucleoli during the G1 cell cycle. Overexpression of SIRT6 leads to slower mitotic process [50]. SIRT6 is also localized in the endoplasmic reticulum, where it deacetylates tumor necrosis factor-α (TNF-α) [53]. SIRT7 is located at the nucleolus and is involved in rRNA transcription mechanism [56]. The main site of SIRT2 is the cytoplasm, but in some phases of the cell cycle it is also found in the nucleus. SIRT2 is responsible for the deacetylation of tubulin microtubules [43,44] and appears to play a key role in adipocyte differentiation [45]. In addition, its action is necessary for the proper separation of chromosomes during mitosis [46] as well as for the exit of cells from this phase [55,56,57].

The isoenzymes SIRT3, SIRT4 and SIRT5 are found in the mitochondria and contribute to oxidative stress alleviation by regulating the activity of specific metabolic enzymes [41] and ATP synthesis, metabolism and intracellular signaling. SIRT3 can be moved between the nucleus and mitochondria under cellular stress [13,41].

### 4.2. Structure and Substrates of SIRTs

**Figure 2:**
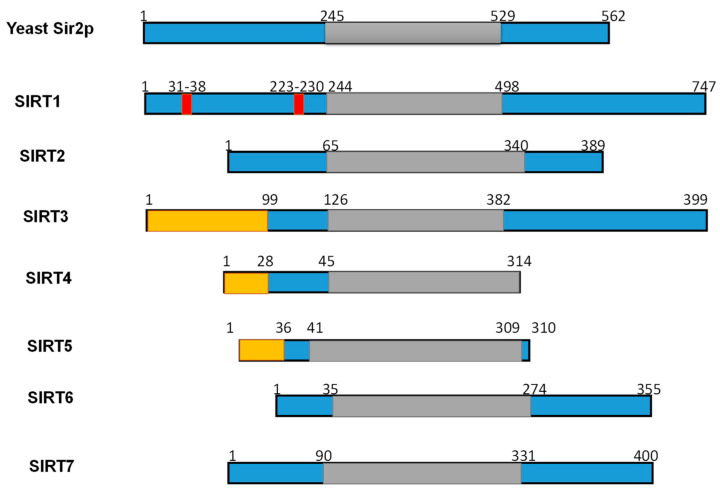
Schematic overview of human SIRTs (SIRT1–7). Human SIRTs are aligned with yeast Sir2p. The conserved large catalytic domain is shown in grey. The nuclear localization sequences shown in red and the mitochondrial targeting sequences are shown in dark yellow. Numbers refer to amino acid residues in the proteins. Adapted from Ref. [9].

**Figure 3:**
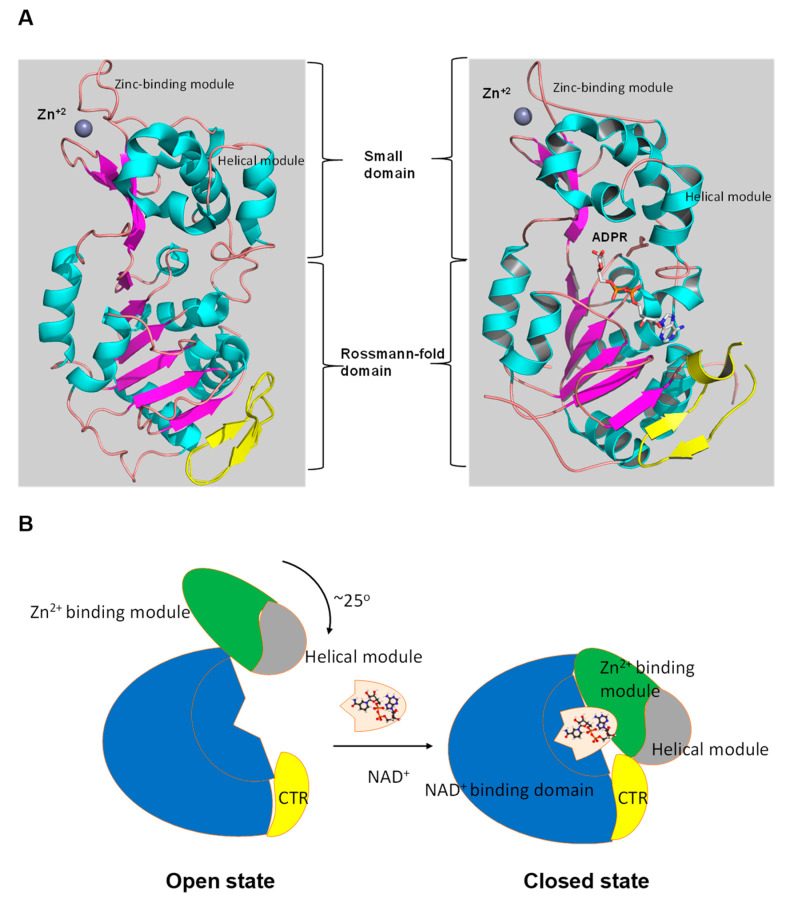
The tertiary structure of SIRT1. (**A**) The structure in open (**left**) conformation (SIRT1•CTR complex, PDB code 4IF6) and in closed (**right**) conformation (SIRT1•CTR•ADPR•Substrate complex, PDB code 4KXQ). The amino acid sequence of SIRT1 is organized into two independent domain. A cavity that is created between the two domains forms the active site. The C-terminal regulatory segment (CTR) is shown in yellow. The CTR binds at the lower edge of the larger NAD^+^-binding domain, complementing the central parallel β sheet of its Rossmann fold. The figure was created using the program PyMOL (www.pymol.org). (**B**) Cartoon model of the conformational changes of SIRT1 upon substrate and NAD^+^ analogue binding. The smaller domain undergoes a rotation with respect to the large domain. A cartoon representation of the apo SIRT1·CTR heterodimer (open state) and the SIRT1·CTR·NAD^+^·Substrate complex (closed state). Comparison of the open and closed structures revealed that the larger NAD+-binding domain does not undergo any major structural changes. The smaller domain rotates about 25°. The small domain (Zn^2+^-binding module and the helical module) rotates as a rigid body with only minor changes to the backbone and sidechain conformations. Adapted from Ref. [62].

Numerous studies have examined the structural features of SIRTs (Figure 2) [18,57,58]. SIRTs composed by approximately 275 amino acids that are organized into two structurally distinct domains: the conserved large domain that has the characteristic structure of the Rossmann fold and the small domain that is less conserved among the members of the SIRT family and contains a zinc ion binding site with the consensus sequence Cys-X2–4-Cys-X15–40-Cys-X2–4-Cys [54,59,60,61,62] and a helical module [59,62] (Figure 3). The Rossmann fold is composed of six parallel β-strands that are grouped in a central β-sheet surrounded by α-helices. The smaller domain consists of three opposite parallel β-sheets where the tetrahedral zinc ion is bound (Figure 3).

**Figure 4:**
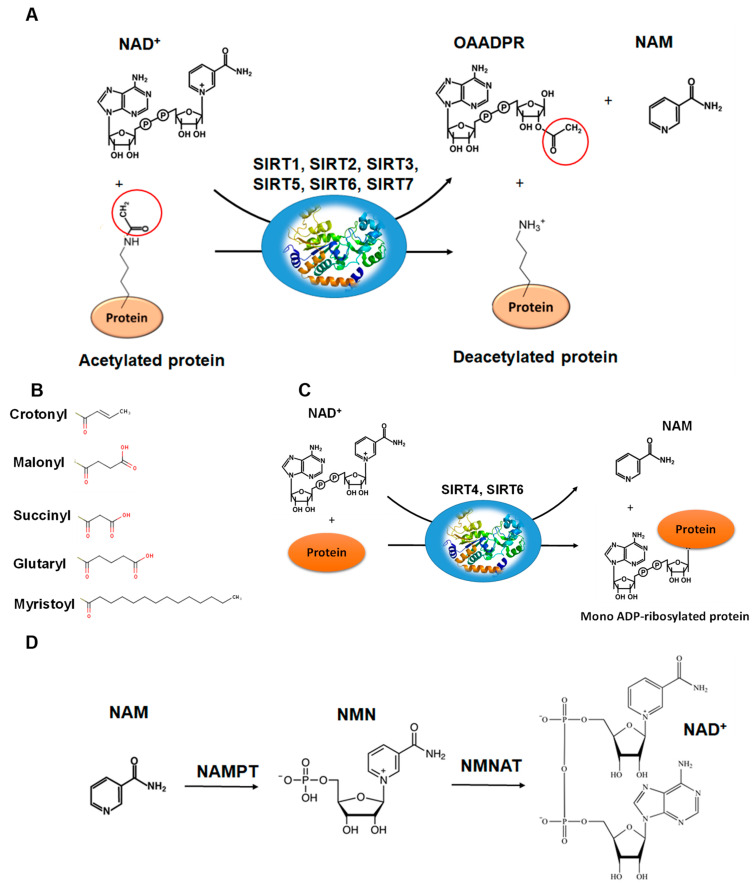
The reactions catalyzed by SIRTs. (**A**) The general deacetylase reaction catalyzed by SIRT1,2,3,5,6,7. The natural substrates are NAD^+^, water and a protein containing acetylated lysine residue. The reaction products include nicotinamide (NAM), the deacetylated protein and the 2’-O-acetyl-ADPR (OAADPR) molecule. (**B**) Alternative acyl groups that can be deacylated by SIRT3,5,7. (**C**) The ADP-rebosyltransferase activity catalyzed by SIRT4 and SIRT6. (**D**) NAM can be used as precursor for the biosynthesis of NAD^+^ by the enzymes nicotinamide phosphoribosyltransferase (NAMPT) and nicotinamide mononucleotide adenylyltransferase (NMNAT).

The deacetylation reaction catalyzed by SIRTs is shown in Figure 4A. The reaction includes three substrates: NAD^+^, water and the acetylated protein. The catalytic cycle involves the formation of the enzyme/NAD^+^/acetylated substrate ternary complex [59,63,64,65]. In the deacetylation process, the glycosidic bond between the nicotinamide and ADP-ribose is cleaved and the free nicotinamide (NAM) is released. Then, the acetyl moiety from the substrate is transferred to ADP-ribose to form acetyl-ADP-ribose (2′-O-acetyl-ADP-ribose, AADPR) and the deacetylated protein [55,63]. The biological role AADPR has not been established so far; however, reasonable experimental results suggest that it acts as a signal transducer [65]. Under normal conditions, AADPR is spontaneously isomerized to 3’-O-acetyl-ADPR. 

The active site of SIRTs is formed by an extended “clef” that is responsible for the recognition and binding of the NAD^+^ cofactor, and is located at the Rossmann fold [63]. The NAD^+^ binding site can be divided into three sub-regions: (a) the adenine and sugar ribose binding site, (b) the nicotinamide ribose binding site, and (c) the nicotinamide moiety binding site. When the acetylated lysine substrate binds to the enzyme, NAD^+^ can undergo a conformational change, bringing the nicotinamide group deeper to the c site where it can be cleaved [59,60,62]. The active site allows the carbonyl oxygen group of acetylated lysine substrate to come into contact with the anomeric carbon of the nicotinamide riboside of the NAD^+^. This effective binding accelerates the reaction of acetyl-oxygen with the anomeric carbon, leading to the cleavage of the nicotinamide moiety of NAD^+^, and the transfer of ADP-ribose to acyl-oxygen, leading to deacetylation.

The deacetylation of lysine residues using as cofactor NAD^+^ remains the most common reaction that describes the catalytic function of SIRTs more accurately, although there are several examples (Table 1) where they can act on non-acetylated substrates [66,67,68] (Figure 4B). For example, it has been reported that succinylated lysine residues in hepatic mitochondria is a target of SIRT5 [50,51]. Another example includes SIRT6, which exhibits demyristoylation activity and has the ability to deacylate long fatty acid aliphatic chains in nuclear factor-κB (NF-κB) factor [55]. SIRT4 acts as an ADP-ribosyltransferase [49] (Figure 4C), whereas SIRT6 exhibits both of these enzymatic activities [60,69]. SIRT4 also shows lipoamidase activity [49] and SIRT5 displays high desuccinylation activity [50,51,52]. 

SIRTs are able to recognize many different protein substrates, although they were originally classified exclusively as histone deacetylases [48,67,68]. For example, SIRT1 has been shown to deacetylate histone H1 on lysine Lys26, H3 on Lys9, Lys14 and Lys56, and H4 on Lys8, Lys12 and Lys16 [67]. The presence of SIRTs in subcellular compartments that do not contain histones prompted the researchers to redefine the range of their protein substrates. Subsequent studies have shown that several other protein substrates are not histones but transcription factors or enzymes that are responsible for cell regulation and adaptation [31,64,68]. 

The enzymatic activity of SIRTs can also be affected by post-translational modifications. Phosphorylation is one of the most well-known mechanisms of post-translational regulation of SIRTs [69]. For example, SIRT1 has fifteen phosphorylation sites and several protein kinases, e.g., c-Jun N-terminal kinases (JNKs), casein kinase 2 (CK2), cyclin dependent kinase 1 (CyclinB/Cdk1), dual-specificity tyrosine-phosphorylation-regulated kinases (DYRKs), have been identified with the ability to phosphorylate these sites and thus affect its activity. The phosphorylation and dephosphorylation status of SIRT1 not only influence the catalytic function of the enzyme itself, but also regulate its expression levels through a protease-dependent or independent degradation mechanism [69]. The catalytic function of SIRT2 is also regulated through phosphorylation/dephosphorylation processes. Phosphorylation leads to enzyme activation, whereas dephosphorylation inhibits its activity.

## 5. The Regulation of SIRTs by NAD^+^ and Natural Products

### 5.1. The Mechanism of SIRT1 Regulation by NAD^+^

The biochemical role of SIRTs and their activity is affected by the availability of NAD^+^ and its intermediates (NADH, NAM) [9,10]. Several studies performed using yeast and human cells indicated that nicotinamide (NAM) and NAD^+^ levels are important regulators of SIRT activity, which in turn are affected by individual cell conditions [70,71,72]. For example, under conditions of caloric restriction or physical exercise, it is known that the action of SIRTs is enhanced [11,15,16]. In these cases, it has been observed that increasing the intracellular level of Ca^2+^, positively affects the metabolic enzyme adenosine monophosphate-activated protein kinase (AMPK) [15,16]. AMPK, in turn, increases NAD^+^ levels through the up-regulation mechanism of nicotinamide phosphoribosyltransferase (NAMPT), which, together with nicotinamide mononucleotide adenyltransferase (NMNAT) are key enzymes for the biosynthesis of NAD^+^ [71,72] (Figure 4D).

**Figure 5:**
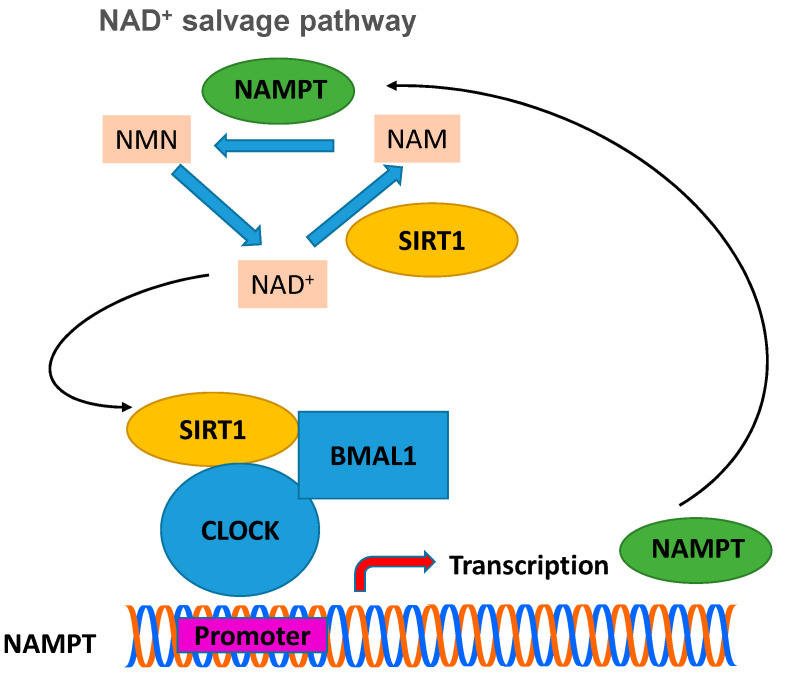
The circadian clock machinery regulates the biosynthesis of NAD^+^ through control of the NAD^+^ salvage pathway. BMAL-1:CLOCK heterodimer binds to the promoter region of NAMPT to regulate the rhythmic transcription of this gene and thus the levels of NAD^+^. Expression of SIRT1 silences the expression of NAMPT (the rate-limiting enzyme in NAD^+^ biosynthesis), which leads to a decrease in the concentration of NAD^+^, diminishing the activity of SIRT1. When the activity of SIRT1 decreases significantly, then the activity of CLOCK:BMAL-1 begins to increase, which restores the expression levels of NAMPT and thus completes the cycle. NAMPT: nicotinamide phosphoribosyltransferase; CLOCK: basic helix-loop-helix-PAS transcription factor; BMAL1: Brain and muscle ARNT-like 1 protein; NMN: nicotinamide mononucleotide; NAM: nicotinamide.

The metabolism and behavior of mammals, including humans, have been found to be regulated by the circadian rhythm [69,73,74,75]. The center for controlling and regulating the circadian rhythm of mammals is located in the neurons of the supraspinatus nucleus of the brain and in cells of the peripheral tissues, where the expression of genes takes place at a periodicity of twenty-four hours. The “molecular oscillator” is regulated by positive and negative transduction signals, which create regression cycles, so that periodic changes (oscillations) are achieved. SIRT1 has been found to function as a key regulator of molecular mechanisms that control circadian rhythm [4,73,74,75,76]. Subsequent studies have shown that intracellular levels of NAD^+^ show periodic daily fluctuations that are fully in line with circadian rhythms. The enzyme NAMPT, which catalyzes the first step in the biosynthesis of NAD^+^ from NAM, requires the presence of SIRT1, which binds to the NAMPT promoter. Subsequent studies provided evidences that the expression levels of NAMPT in mammals show periodic fluctuations [75]. The heterodimeric transcriptional activator of the circadian rhythm, CLOCK (basic helix-loop-helix-PAS transcription factor):BMAL-1 (brain and muscle ARNT-like 1 protein), induces the expression of NAMPT, whose action is inhibited by SIRT1 (see Figure 5) [75,77,78]. In short, expression of SIRT1 silences the expression of NAMPT, which leads to a decrease in the concentration of NAD^+^, diminishing the activity of SIRT1. When the activity of SIRT1 decreases significantly, then the activity of CLOCK:BMAL-1 begins to increase, which restores the expression levels of NAMPT and thus completes the cycle [75,76].

### 5.2. Natural Products as SIRT1 Modulators

**Figure 6:**
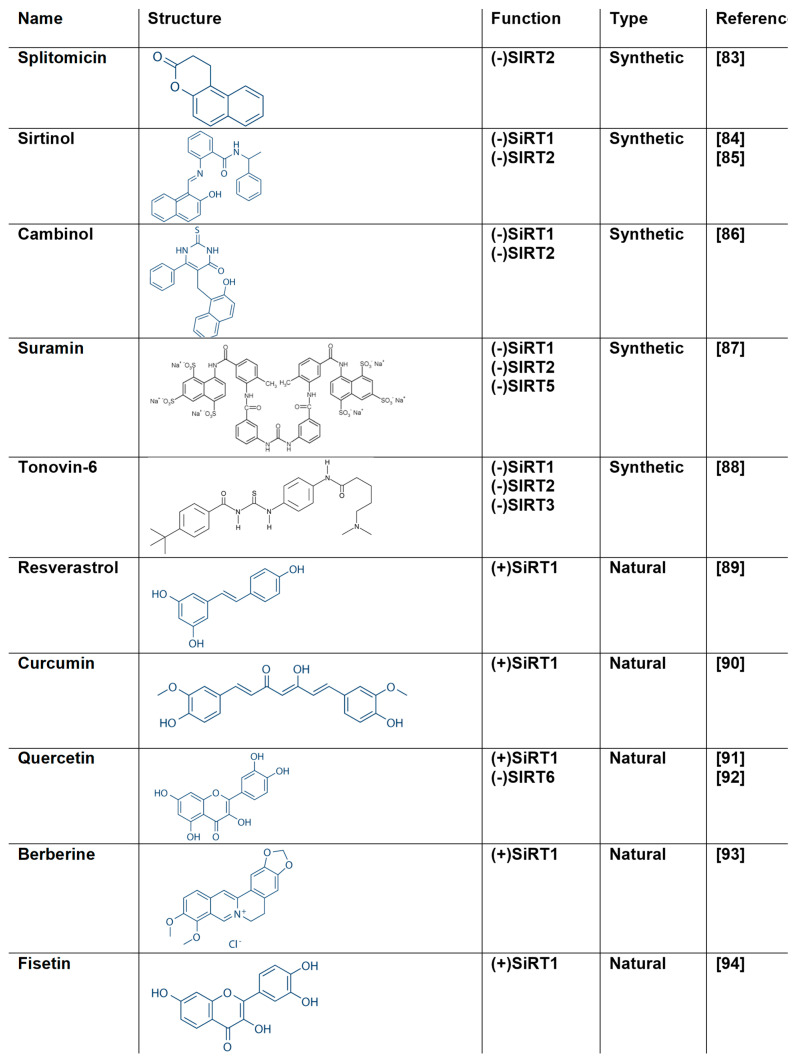
The structure of natural and synthetic SIRT modulators [83,84,85,86,87,88,89,90,91,92,93,94].

Natural products chemistry continues to be an excited area for discovering new drugs or lead compounds [26,28]. The diversity and complexity of natural products can provide excellent source for bioactive molecules with remarkable efficacy and specificity. Therefore, they hold a great potential for new scaffolds discovery with the ability to modulate therapeutic protein targets [41,78,79]. SIRT modulators are compounds that are able to inhibit or activate SIRTs’ activity (Figure 6). They gained a particular interest as they can regulate (activate or inhibit) their function [60,61,62,78,79,80,81]. For example, SIRT1 activators have been proposed for treating a range of diseases and disorders such as aging, oxidative stress, diabetes, obesity, neurodegenerative diseases, cardiovascular disease and inflammation [78,79,80,81,82].

Studies related to metabolic disorders and cancer suggest that in addition to activators, finding inhibitors of SIRT1 could be equally useful. Both antagonistic mechanisms, i.e., SIRT1 activation and inhibition, have been proposed in cancer therapy [12,82,83,84]. SIRT1 inhibition has also been proposed in the treatment of virus infections [71], whereas SIRT2 inhibitors might be useful for the treatment of cancer and neurodegenerative diseases [80,81,83]. Therefore, the discovery of small molecules that display a combination of activating and inhibitory activity across the seven different SIRTs, with the mode of action of each compound tailored to treat different diseases, is highly relevant.

To date, several modulators with a wide range of core structures have been identified and characterized [41,79]. Some well-known modulators of SIRTs are shown in Figure 6 and briefly discussed. Splitomicin inhibits SΙRΤ2 deacetylase activity with an IC50 of 60 μM by altering or blocking the access to the acetylated histone binding pocket [83]. Sirtinol is a specific SIRT1 and SIRT2 inhibitor with IC50 131 μM and 38 μM, respectively [84,85]. Cambinol is an inhibitor for both SIRT1 and SIRT2 with IC50 values 56 and 59 μM, respectively [86]. Cambinol has the same β-naphthol moiety with sirtinol and splitomicin. Suramin is a polyanionic naphthylurea and strong inhibitor of SIRT1 and SIRT2 with IC50 values 0.297 μM and 1.15 μM, respectively. Suramin is also a weaker inhibitor for SIRT5 (IC50 22 μM) [87]. Several viruses have been described to be inhibited by suramin including HIV, HSV-1, HBV, HCV. Tenovin-6 inhibits the protein deacetylase activities of purified human SIRT1, SIRT2 and SIRT3 in vitro with IC50 of 21 μM, 10 μM and 67 μM, respectively [88]. 

The most well-known modulator of SIRTs is resveratrol (3,5,4-trihydroxy-stilbene), which is the first SIRT1 activator that was identified [89] (Figure 6). It belongs to the phytoalexins and is present at high concentrations in grapes, eucalyptus and fir leaves, nuts and berries, while in lower concentrations it is found in many other plants [95]. It displays remarkable antioxidant activity and several studies have proven its anti-cancer [96], anti-inflammatory and anti-aging activities [97,98]. 

**Figure 7:**
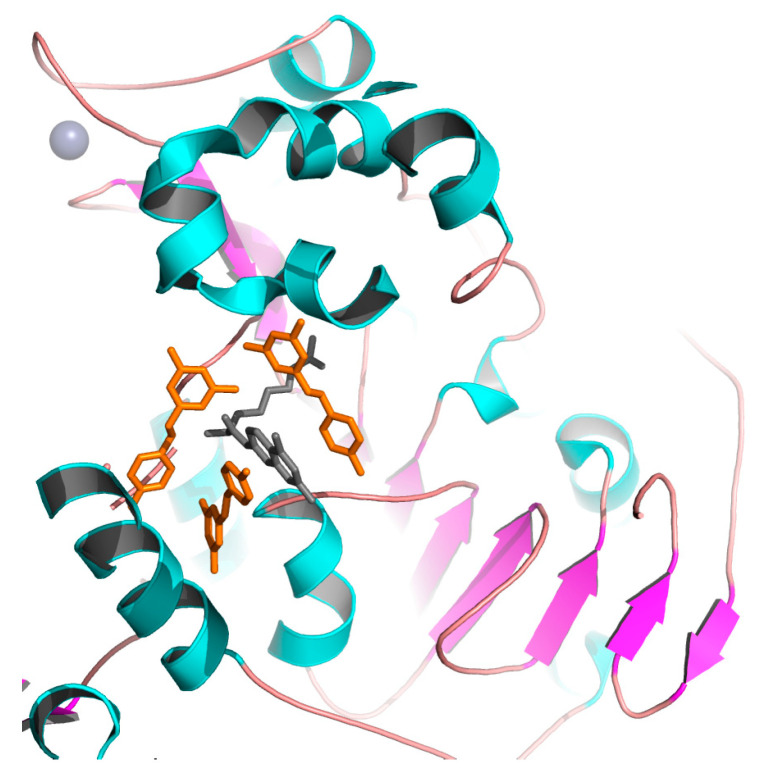
Crystal structure of human SIRT1 in complex with resveratrol (shown in orange) and 7-amino-4-methylcoumarin containing peptide (shown in grey). The zinc ion is shown as a grey sphere. The figure was created using the program PyMOL (www.pymol.org).

Resveratrol was first described as a SIRT1 activator. Howitz et al., 2003, demonstrated that resveratrol was capable of reducing the K_m_ of both the acetylated substrate and NAD^+^ (35- and 5-fold, respectively) [8]. Hubbard et al., showed that resveratrol is an allosteric activator of SIRT1 [99]. X-ray crystallography was used to determine the structural basis of substrate-dependent activation of SIRT1 by resveratrol [100,101] (Figure 7). Analysis of the crystal structure of SIRT1 in complex with resveratrol and 7-amino-4-methylcoumarin (AMC)-containing peptide substrate showed that two resveratrol molecules were bound at the Rossmann fold domain of SIRT1, interacting with the peptide substrate. This interaction promotes a tighter binding between SIRT1 and AMC peptide, and thus stimulates SIRT1 activity. Resveratrol also promotes the activation of SIRT5 and the weak inhibition of SIRT2 and SIRT3 [102]. Intense research effort focuses on the design and synthesis of more potent and efficient resveratrol-like analogues with increased bioavailability [103,104]. Some of them appeared to have a stronger effect, compared to resveratrol, as for example, the tri-acetyl-stilbene that was found to be more effective in prolonging life.

Although initially the role of resveratrol as a direct activator of SIRT1 was criticized, recent mechanistic data demonstrates that, at least indirectly, resveratrol activates SIRT1 activity in vivo by increasing NAD^+^ available concentration [105]. Another mechanism that can shine light on the protective effect of resveratrol connects the increase in SIRT1 activity, which enhances the deacetylation of forkhead box protein O1 (FOXO1) and the activation of manganese superoxide dismutase (MnSOD) downstream. The induction of MnSOD alleviates oxidative stress [106,107]. Han et al., in 2020 showed that resveratrol reduces hypoxia-induced apoptosis in H9C2 cells through the activation of SIRT1/miR-30d-5p/NF-kB axis [108].

Curcumin [1,7-bis(4-hydroxy-3-methoxyphenyl)-1,6-heptadiene-3,5-dione] (Figure 6), a polyphenol derived from the turmeric plant, is another well-studied natural product that activates SIRT1 [90]. The flavonoid polyphenol quercetin, 3,3,4,5,7-pentahydroxyflavone, is a natural glycoside that has antioxidant and anti-inflammatory properties [91,92,109]. Quercetin is believed to act via the AMPK/SIRT1 signaling pathway [110]. 

Berberine (Figure 6) is an isoquinoline alkaloid reported to have analgesic, anticancer, anti-inflammatory and myocardial protective properties [111]. Berberine is an activator of SIRT1 and through this mechanism, is able to decrease FOXO1 acetylation, triggering anti-apoptotic signaling pathways via Bcl-2 expression, Bax and caspase-3 downregulation [93]. The dietary flavonoid fisetin (3,3,4,7-tetra-hydroxyflavone) (Figure 6) can counteract oxidative stress and mediate immune response via AMPK/SIRT1 and Nfr2 pathways [92]. Fisetin was shown to increase SIRT1 expression and enhance SIRT1-mediated peroxisome proliferator-activated receptor (PPAR) and FOXO1 deacetylation in 3T3L1 cells [94]. 

Chalcones have shown inhibitory properties against SIRT1. Kahyo et al., 2008 showed that the chalcone derivative 3,20,30,40-tetrahydroxychalcone displayed inhibitory potency against the SIRT1-mediated deacetylation of a p53 acetylated peptide [112]. Bichalcones have also been shown to be potential SIRT inhibitors. For example, the bichalcone rhuschalcone I isolated from the medicinal plant Rhus pyroides Burch showed inhibitory activity against SIRT1 with an IC50 value of 40.8 μM [113].

## 6. SIRTs as Emerging Antiviral Targets

### 6.1. The Role of SIRTs in Viral Infections

The unexpectedly rapid emergence of SARS-CoV-2 has raised a great concern that a pandemic could spread rapidly without time to prepare a public health response to stop the illness spread [114]. As we are still unable to predict with confidence the progress of COVID-19 pandemic and considering that the next pandemic is most likely to be caused by influenza, the discovery of antiviral compounds has to be the priority public health threat in the world [115,116]. Most importantly, we need to have a range of available antiviral tools that can respond rapidly and effectively to emergencies.

Viruses depend on host-cell metabolism for energy, for production of viral components and genomes, as well as for organization of cellular compartments of replication, maturation and dissemination. As such, the control of the host cell’s metabolism by SIRTs appears to be an essential component that regulates the viral-host interaction [117,118,119,120,121]. Taking into account that SIRTs are molecular targets on human cells rather than on viruses, the development of resistance is less likely to occur. Several members of SIRTs have been previously shown to affect a broad range of viral pathogens [122,123,124,125]. It has been reported that, in some cases, SIRTs promote infection, while in other cases, SIRTs restrict infection [117,122,123,126,127,128,129,130,131]. Given the diverse activities of SIRTs as key regulators of transcription and metabolism, they can be considered as effective antiviral targets for the development of broad-spectrum antivirals, similar to the broad-spectrum antibiotics [119,120,121]. In addition, SIRTs can affect the replication of DNA and RNA viruses; hence, targeting through inhibition or activation can provide an effective antiviral therapeutic strategy. 

The regulation of NAD^+^ intercellular level through the inhibition/activation of SIRTs is presumably one approach [118,132]. This is supported by the observation that certain viruses have already developed this capability. For example, it has been reported that HSV-1 infection leads to reduced NAD^+^ levels [133]. The balance between oxidized and reduced forms of NAD^+^ is an important component of the redox state of a cell, a balance reflecting both the metabolic activities and the status of the cell. In addition, the stress caused by viral replication often establishes a high NAD^+^-state that activates SIRTs [128,132]. Another approach can be based on the transcriptional functions of certain SIRTs that appear necessary for the regulation of viral gene expression [120,121,134,135]. Therefore, fine-tuning of SIRT regulation at the level of one enzyme or one function may be a valuable way forward to take advantage of SIRT defense properties [120,135,136].

### 6.2. SIRT1 and SIRT2 Inhibitors Can Be an Option to Treat Viral Infections, Including COVID-19

SIRT1 inhibitors affect the replication and growth of many viruses including Mers-CoV, HIV, Epathitis B, Vesicular Stomatitis Virus, flu strains, adenovirus and others [126,135,136,137,138,139]. Notably, SIRT1 affects angiotensin-converting enzyme 2 receptor (ACE2R) expression [140,141,142]. The ACE2R plays the role of the host cell receptor for the virus and binds through the spike protein on the viral capsid [143]. This is a key event for infectivity, activating the clathrin-dependent endocytosis of both the ACE2R and virus. Inhibition of SIRT1 activity can affect and reduce the expression of ACE2, as the expression of the ACE2 transcript is controlled by the activity of SIRT1 under conditions of energy stress [141]. 

Energy stress lowers the available level of NAD^+^. NAD^+^ level declines with age and is also reduced in conditions associated with oxidative stress. Koyuncu et al., (2014) have reported that small interfering RNA (siRNA) knockdown for each of the seven SIRT genes enhances the growth of several viruses, similar to that observed after treatment with a SIRT1 inhibitor [123]. On the other hand, compounds that activate SIRT1, prevent the production of viral progeny. Interestingly, similar response was observed after knockout or overexpression of the *Escherichia coli* SIRT, CobB, which regulates the growth of bacteriophages, suggesting that SIRTs can be considered as broad-spectrum, evolutionarily conserved viral restriction factors [123]. 

The life-cycle of a virus involves the biosynthesis of viral components that depend on host-cell metabolic conditions. Therefore, the regulation of the host cell’s metabolism is a key function of the viral-host interaction [117,118,119,120,121]. One of the core roles of SIRT1 and SIRT2 is to control metabolism and gene expression through post-translational modification of several regulatory proteins in the host cell as well as in virus [19,41]. Through these mechanisms, SIRTs may be able to control the outcome of viral infection by regulating both host and viral gene expression. Two examples include the interaction of SIRT1 and SIRT2 with the protein p53 (cellular tumor antigen p53) and the transcription factor c-MYC, respectively. It is well established that p53 is a protein substrate for SIRT1 and is deacetylated in a NAD^+^-dependent manner, leading to the inhibition of its transcription activity and the modulation of pathways that are implicated in regulation of tissue homoeostasis [144,145,146,147]. When a cell is subjected to stress, such as during viral infection, p300 acetylates and activates p53, triggering a host process that inhibits viral replication, leading to infected cell apoptosis. However, some viruses have evolved mechanisms for deacetylation of p53—such as up-regulation of SIRT1 and SIRT2—that render p53 inactive, allowing the cell to survive and the virus to propagate [148]. Inhibition of SIRT1 can block viral-induced deacetylation of p53, causing hyperacetylated p53 (accumulation of active p53), leading to cell death and virus elimination. SIRT2 inhibition leads to the degradation of the transcription factor c-MYC via induction of the ubiquitin ligase NEDD4 [149]. Many viruses such as adenovirus, herpes simplex virus 1 and influenza A upregulate c-MYC to activate the genes that are required for glutamine utilization, which is consumed for viral nucleic acid biosynthesis [150,151].

**Figure 8:**
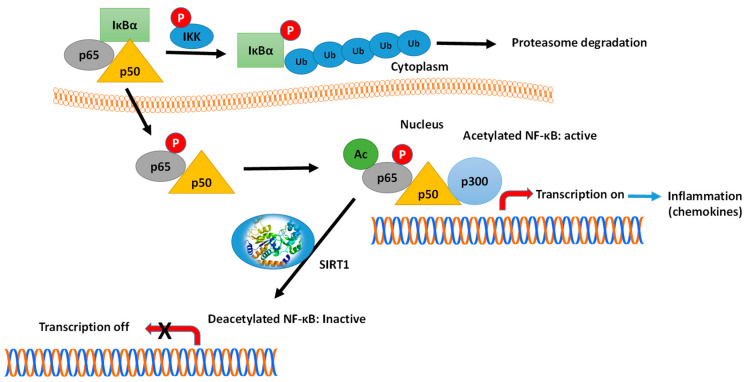
Mechanism of NF-κB action and the role of SIRT1. In the inactivated state, the heterodimer NF-κB, composed by p65 (RelA) and p50 proteins, is located in the cytosol complexed with the inhibitory protein IκBα. A variety of extracellular signals can activate the enzyme IκB kinase (IKK), which phosphorylates the IκBα protein, leading to dissociation of the inhibitory protein IκBα from NF-κB. The phosphorylated IκBα is subjected to ubiquitination, leading to its degradation by the proteasome. The activated NF-κB is then translocated into the nucleus and interacts with specific sequences of DNA. The DNA/NF-κB complex then binds to coactivators (e.g., p300-CBP) and RNA polymerase, which transcribes downstream DNA into mRNA. SIRT1 suppresses NF-κB transcription factor by deacetylation of the p65 (RelA) subunit. Acetylation of NF-κB increases the transcription of proinflammatory mediators. Activators of SIRT1 can lead to repression of inflammation.

Another example is the interaction of SIRTs with key transcription factors, such as NF-κB and FOXO1 [152,153,154,155]. The connection and crosstalk between NF-κB and SIRT1 in the regulation of inflammation (see Figure 8) and metabolic disorders have been investigated thoroughly by Kauppinen et al. [152]. Various subunits of the NF-κB family of transcription factors are acetylated/deacetylated at multiple sites, affecting the DNA-binding and transcriptional activity of these proteins. For example, the p65(RelA) subunit is deacetylated at Lys310 by SIRT1, causing the inhibition of the NF-κB-mediated signaling [156,157]. Kauppinen et al., suggested that NF-κB signaling plays a key function in innate immunity defense, while SIRT1 controls the oxidative respiration and cellular survival [152]. On the other hand, NF-κB signaling down-regulates SIRT1 activity through the expression of miR-34a, IFNγ and reactive oxygen species. The inhibition of SIRT1 disrupts oxidative energy metabolism and stimulates the NF-κB-induced inflammatory responses. Hariharan et al., have reported that the SARS-CoV-2 components induce the activation of NF-κB in different cells, leading to the production of various chemokines (chemokines ‘storm’) [153] (see Figure 8).

Another mechanism that can be taken into account involves the activation of Nod-like receptor family pyrin domain containing-3 inflammasome (NLRP3) caused by the upregulation of SIRT2 [158,159]. In older individuals, NLRP3 may be poised for hyperactivation by SARS-CoV-2 components. The modulation of NLRP3 activity is under the direct control of SIRT2 [160]. Old mice, especially those deficient in SIRT2, have accelerated inflammaging, along with decreased glucose tolerance and increased insulin resistance. During aging, NAD^+^ levels decline, reducing the activity of SIRTs [161,162,163,164]. This decrease might give rise to hyperactivation of NLRP3 and the trigger cytokine storms [165]. Maintaining NAD^+^ levels through the SIRTs system may therefore alleviate COVID-19 symptoms, a possibility supported by recent data showing that SARS-CoV-2 proteins hyperactivate poly-ADP-ribose polymerases (e.g., PARP9, -10, -12, and -14) and deplete cellular NAD^+^ [162,163,164,165,166,167]. The ability of NAD^+^ precursors to lower inflammation in human subjects provides further support to this mechanism [163,164]. These mechanisms probable interpret the observation that elderly patients with sensitized NF-κB and metabolic syndrome are very susceptible to COVID-19 with worse complications and high mortality. Presumably, the inhibition of SIRT1/NF-κB pathway has a therapeutic role in alleviating the severe form of COVID-19.

## 7. Conclusions

SIRTs offer a remarkably rich diversity of regulatory points. Their NAD^+^-dependent activities allow them to transmit information about changes in the environment to major cellular pathways for rapid and effective responses. A growing body of evidences suggest that SIRTs have key roles in the field of virology. The diversity and abundance of SIRT substrates complicate the interpretation of their roles during infection. This illustrates the need for further research for gaining deeper insight into the SIRT-mediated events during viral infections.

## Figures and Tables

**Table 1:** The class, functional activity, molecular mass and chromosomal location of seven human SIRTs (SIRT1–7).

Sirtuins	Class	Functional Activity	Molecular Mass (kDa)	Chromosomal Location	References
SIRT1	I	Deacetylase, Deacylase	81.7	10q21.3	[17,18,41,42]
SIRT2	I	Deacetylase, Deacylase	41.5	19q13.3	[8,43,44,45,46]
SIRT3	I	Deacetylase, Decrotonylase	43.6	11p15.5	[13,17,18,41,47,48]
SIRT4	II	Deacetylase, ADP-ribosyltransferase, Lipoamidase, Deacylase	35.2	12q	[17,18,49]
SIRT5	III	Deacetylase, Desuccinylase, Demalonylase, Deglutarylase	33.9	6p23	[21,22,23,50,51,52]
SIRT6	IV	Deacetylase, Demyristoylase, ADP-ribosyltransferase, Deacylase	39.1	19p13.1	[18,20,47,53,54,55]
SIRT7	IV	Deacetylase, Desuccinylase,	44.9	17q25	[18,56]
